# Topography of FUS pathology distinguishes late-onset BIBD from aFTLD-U

**DOI:** 10.1186/2051-5960-1-9

**Published:** 2013-05-09

**Authors:** Edward B Lee, Jenny Russ, Hyunjoo Jung, Lauren B Elman, Lama M Chahine, Daniel Kremens, Bruce L Miller, H Branch Coslett, John Q Trojanowski, Vivianna M Van Deerlin, Leo F McCluskey

**Affiliations:** 1 Perelman School of Medicine at the University of Pennsylvania, Philadelphia, PA 19104, USA; 2 Translational Neuropathology Research Laboratory, 605B Stellar Chance Laboratories, 422 Curie Blvd, Philadelphia, PA 19104, USA; 3 Department of Pathology and Laboratory Medicine, Philadelphia, PA, USA; 4 Center for Neurodegenerative Disease Research, Philadelphia, PA, USA; 5 UCSF Department of Neurology, Memory and Aging Center, San Francisco, CA, USA; 6 Department of Neurology, Philadelphia, PA, USA; 7 Department of Neurology, Jefferson Medical College of Thomas Jefferson University, Philadelphia, PA, USA

**Keywords:** Frontotemporal dementia, Frontotemporal lobar degeneration, Motor neuron disease, Amyotrophic lateral sclerosis

## Abstract

**Background:**

Multiple neurodegenerative diseases are characterized by the abnormal accumulation of FUS protein including various subtypes of frontotemporal lobar degeneration with FUS inclusions (FTLD-FUS). These subtypes include atypical frontotemporal lobar degeneration with ubiquitin-positive inclusions (aFTLD-U), basophilic inclusion body disease (BIBD) and neuronal intermediate filament inclusion disease (NIFID). Despite considerable overlap, certain pathologic features including differences in inclusion morphology, the subcellular localization of inclusions, and the relative paucity of subcortical FUS pathology in aFTLD-U indicate that these three entities represent related but distinct diseases. In this study, we report the clinical and pathologic features of three cases of aFTLD-U and two cases of late-onset BIBD with an emphasis on the anatomic distribution of FUS inclusions.

**Results:**

The aFTLD-U cases demonstrated FUS inclusions in cerebral cortex, subcortical grey matter and brainstem with a predilection for anterior forebrain and rostral brainstem. In contrast, the distribution of FUS pathology in late-onset BIBD cases demonstrated a predilection for pyramidal and extrapyramidal motor regions with relative sparing of cerebral cortex and limbic regions.

**Conclusions:**

The topography of FUS pathology in these cases demonstrate the diversity of sporadic FUS inclusion body diseases and raises the possibility that late-onset motor neuron disease with BIBD neuropathology may exhibit unique clinical and pathologic features.

## Background

Since the discovery of missense mutations in the fused-in-sarcoma (*FUS*) gene that are pathogenic for familial amyotrophic lateral sclerosis (ALS), a variety of clinically and pathologically diverse neurodegenerative diseases have been found to demonstrate FUS-positive inclusions in central nervous system (CNS) neurons [1-18]. Rare juvenile and adult onset forms of ALS exhibit basophilic inclusions which are immunoreactive for FUS protein, sometimes but not always associated with *FUS* mutations [3,4,6-8,10,11,13,14,18]. In addition to ALS, rare forms of frontotemporal lobar degeneration (FTLD) also exhibit tau-negative, TDP-43 negative, FUS-positive inclusions in the absence of *FUS* mutations [1,2,5,9,11,14-17]. FTLD is a general pathologic term for a group of heterogeneous diseases characterized neuropathologically by progressive neurodegeneration with a predilection for frontal and temporal lobes and clinically by frontotemporal dementia (FTD) with or without motor neuron disease (MND). The current classification of FTLD variants with underlying FUS pathology combines three entities formerly known as atypical FTLD with ubiquitinated inclusions (aFTLD-U), basophilic inclusion body disease (BIBD) and neuronal intermediate filament inclusion disease (NIFID) into an umbrella category of FTLD with FUS inclusions now known as FTLD-FUS [12].

aFTLD-U cases show characteristic tau-negative, TDP-43-negative and FUS-positive inclusions [9,11,15,17]. aFTLD-U cases are generally sporadic, early-onset, and present clinically as aggressive forms of behavioral variant FTD with prominent psychobehavioral symptoms. Inclusions in aFTLD-U exhibit a variety of morphologies including dystrophic neurites (DN), numerous types of neuronal cytoplasmic inclusions (NCI), and characteristic neuronal intranuclear inclusions (NII) [9,11,15,17]. While aFTLD-U cases may exhibit a few basophilic inclusions in subcortical regions, BIBD exhibits numerous intraneuronal basophilic inclusions on hematoxylin and eosin (H&E) stained sections that are FUS positive [3-6,11,13,14]. In addition to these basophilic inclusions, FUS immunohistochemistry of BIBD brain tissue reveals FUS-positive NCIs in a wide anatomic distribution including the neocortex and hippocampus. NIIs are rare to absent in BIBD and unlike aFTLD-U and NIFID these rare NIIs do not exhibit vermiform morphology [11,14]. The clinical phenotype of BIBD is varied ranging from pure ALS without dementia (i.e. the aforementioned juvenile- or adult-onset cases of sporadic ALS with basophilic inclusions) to ALS with dementia to pure FTD. NIFID is a rare disorder with varied clinical manifestations in which inclusions tend to be eosinophilic and are immunoreactive for class IV neuronal intermediate filaments and FUS [1,2,5,9,11,16,19].

Recent clinicopathologic series of various FTLD-FUS cases reported distinct pathologic features of aFTLD-U, BIBD and NIFID thereby supporting the notion that these three diseases represent related but distinct pathologic entities [5,9,11]. To further highlight the diversity of FTLD-FUS subtypes, we report here the clinical and pathologic findings of three cases of aFTLD-U and two cases of late-onset BIBD which have not been previously described in detail. Sampling throughout the neuraxis demonstrated that the topographic distribution of FUS pathology was distinct between these few cases aFTLD-U and late-onset BIBD, suggesting that although they share a common molecular pathology (i.e. FUS positive inclusions), there is considerable heterogeneity amongst the sporadic FUS inclusion body diseases. Furthermore, the two late-onset BIBD cases exhibit clinical and pathologic features that are distinct from most reported cases of BIBD suggesting that late-onset sporadic ALS with BIBD neuropathology may represent an extreme example on one end of the diverse spectrum of sporadic FUS inclusion diseases.

## Results

Five cases with FUS neuropathology including three cases of aFTLD-U and two cases of BIBD were identified from the greater than 1500 cases in the University of Pennsylvania CNDR Brain Bank. As summarized in Table 1, this included three cases of aFTLD-U (designated as cases F1-3) and two cases of BIBD (designated as cases B1 and B2). The aFTLD-U patients were clinically diagnosed with behavioral variant FTD with age of onset between 42 to 47 years. Cases F2 and F3 also exhibited evidence of parkinsonism and MND. The BIBD cases B1 and B2 were clinically diagnosed with amyotrophic lateral sclerosis-plus syndrome (ALS-plus) with a late age of onset of 65 and 75 years. Case B1 showed no evidence of parkinsonism or cognitive dysfunction but developed diffuse chorea. Case B2 exhibited both parkinsonism and FTD.

**Table 1:** Clinical features of aFTLD-U and BIBD cases

**Case**	**Clinical diagnosis**	**Additional clinical features**	**Autopsy diagnosis**	**Brain weight (g)**	**Age of onset**	**Age of death**	**Gender**	**Clinical synopsis**
F1	bvFTD		aFTLD-U	1130	42	48	F	Inappropriate behavior, loss of interests, obsessive compulsive behaviors, decreased language output, hyperorality. MMSE at presentation 24/30. EMG normal.
F2	bvFTD	Parkinsonism and early MND	aFTLD-U	1200	47	56	M	Inappropriate affect with diminished social skills and aggression, decreased language output. Hyperorality and compulsive behaviors. Motor exam showed mild parkinsonism (increased tone and mild bradykinesia). MMSE at presentation 27/30. EMG with early motor unit dropout.
F3	bvFTD	Parkinsonism and MND	aFTLD-U	1241	46	51	M	Change in personality with progressive apathy, loss of interests, disinhibition with inappropriate laughter, decreased and slow language output, obsessive compulsive tendencies. Motor exam initially showed mild rigidity and brisk deep tendon reflexes without weakness, atrophy or fasiculations. Later developed Hoffman reflexes and pathologically brisk deep tendon reflexes in all four extremities, atrophy of intrinsic hand muscles and tongue, and stiff/slow gait
B1	PMA variant of ALS-Plus	Chorea	BIBD	1229	65	72	F	Progressive gait disorder, arm/hand weakness, muscle atrophy, dysarthria. Subsequent choreaform movements of head/neck with milder involvement of limbs/trunk. No cognitive dysfunction. EMG showed denervation and fibrillations. Died of neuromuscular respiratory failure.
B2	ALS-Plus	Parkinsonism and FTD	BIBD	1569	75	78	M	Progressive weakness with parkinsonian gait, tremor of right hand, micrographia. Logopenic, poor oral trails, only producing three words beginning with the letter “f” in one minute, MMSE initially 28/30 with only one out of three words for delayed recall task. EMG showed denervation and fibrillations. Developed muscle atrophy, fasiculations. Died of neuromuscular respiratory failure.

### Gross and histologic findings

All three aFTLD-U cases showed gross cerebral atrophy affecting the frontal lobe, temporal lobe, and the caudate nucleus. Depigmentation of the substantia nigra was also observed. Histologic examination confirmed the presence of neurodegeneration most profoundly affecting the frontal neocortex, orbitofrontal cortex, temporal cortex, cingulate gyrus, amygdala, hippocampus (with hippocampal sclerosis in cases F1 and F2), parahippocampal gyrus, basal ganglia (caudate nucleus and globus pallidus) and substantia nigra. Inclusions were not appreciated on H&E stained sections aside from a rare nigral basophilic inclusion in case F3.

**Figure 1:**
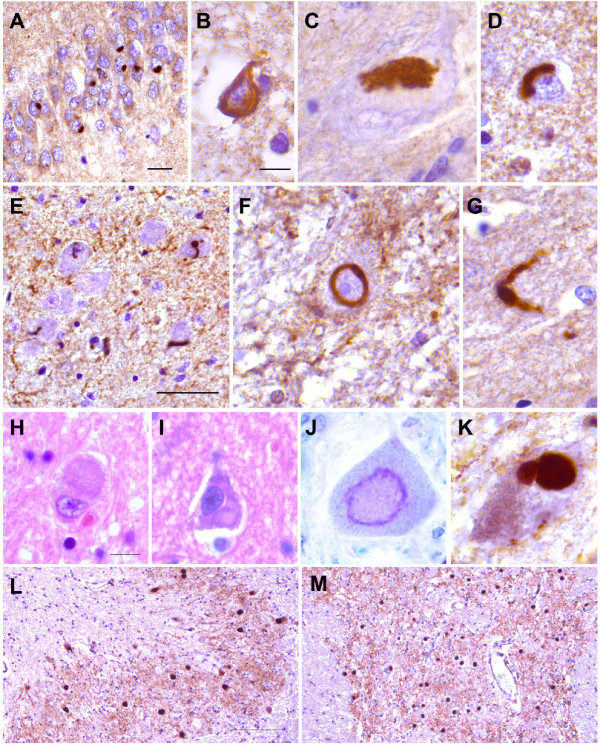
**Morphology of FUS inclusions in aFTLD-U and late-onset BIBD.** Images of representative FUS inclusions from (**A**-**G**) aFTLD-U and (**H**-**M**) BIBD cases are shown. FUS IHC of aFTLD-U revealed various aggregates including (**A**) compact round NCIs in the dentate gyrus of the hippocampus, (**B**) tangle-like inclusions in frontal neocortex, (**C**) a conglomerate aggregate in a substantia nigra neuron, (**D**) perinuclear crescentic inclusions in frontal neocortex, (**E**) numerous vermiform NIIs in the medullary olive nucleus, (**F**) a ring shaped NII in a cerebellar dentate nucleus neuron and (**G**) a swollen DN in the entorhinal cortex. H&E stain of BIBD cases revealed basophilic inclusions including aggregates involving (**H**) the cerebellar dentate nucleus and (**I**) the primary motor cortex. Cresyl violet stain also stained BIBD inclusions including (**J**) an aggregate within a spinal motor neuron. FUS immunohistochemistry of BIBD cases revealed NCIs of various morphologies including (**K**) conglomerate aggregates within a substantia nigra neuron and round NCIs involving the (**L**) dentate nucleus of the cerebellum and (**M**) pontine nuclei. Scale bars for (**A**) 20 μm, (**B**-**D**, **F**-**G**) 10 μm, (**E**) 50 μm, (**H**-**K**) 10 μm and (**L**-**M**) 200 μm are shown.

BIBD showed mild to no gross cerebral atrophy. Histologic examination demonstrated severe loss of spinal motor neurons. Corticospinal tract degeneration was not appreciated in case B1 but was severe in case B2. Case B1 also demonstrated neurodegeneration most prominently affecting the dorsal midbrain, locus ceruleus, substantia nigra, and globus pallidus. Case B2 showed mild to no neuronal loss outside of the pyramidal motor system. In both cases, basophilic to achromatic NCIs were readily identified on H&E or cresyl violet stained sections involving multiple brain regions including the primary motor cortex, ventral spinal cord, inferior olive, dentate nucleus of the cerebellum, basis pontis, substantia nigra and globus pallidus (Figure 1 and data not shown).

Immunohistochemical stains for β-amyloid, tau protein, TDP-43 and α-synuclein together with thioflavin S stains performed on all five cases excluded other neurodegenerative diseases including AD, FTLD with TDP-43 or tau inclusions, and Lewy body disorders. Neurofibrillary tangles were only observed in cases F2, B1 and B2 corresponding to Braak stage I, II and III, respectively. Neuritic plaques were absent in all cases except for rare neuritic plaques in the cingulate and angular gyri of case B1.

### FUS Inclusion morphology

FUS immunohistochemistry was performed on sections throughout the neuraxis to compare the morphology and topography of FUS inclusions in aFTLD-U versus BIBD. As previously described by others, FUS IHC of the aFTLD-U cases studied here revealed inclusions with various morphologies. NCIs exhibited morphologies ranging from compact round inclusions (Figure 1A), to tangle-like inclusions (Figure 1B), larger conglomerate inclusions (Figure 1C), and crescentic perinuclear aggregates (Figure 1D). Abundant numbers of NIIs could be identified including characteristic vermiform (Figure 1E) or circular NIIs (Figure 1F). DNs of various lengths including longer DNs with apparent swollen morphology were also present (Figure 1G). In contrast, FUS inclusions in BIBD were nearly all cytoplasmic and included annular or crescentic NCIs, compact round NCIs, larger conglomerate NCIs and occasional tangle-like NCIs (Figure 1H-M). A single spinal NII was identified in case B2, while NIIs were not seen in case B1. Inclusions in all cases did not stain with antibodies that recognize neurofilament protein, thereby ruling out the possibility of NIFID (data not shown). However, inclusions in all cases did stain with antibodies that recognize ubiquitin, transportin 1, TAF15 and EWS consistent with the absence of pathogenic *FUS* mutation (data not shown). Sequencing of the FUS gene revealed no pathogenic *FUS* mutations in these five cases.

### FUS Inclusion topography

**Figure 2:**
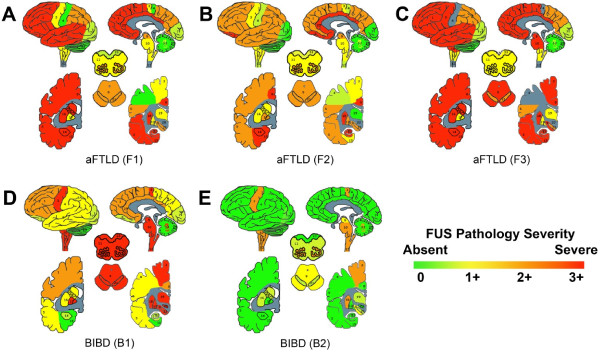
**Topography of FUS inclusions in aFTLD-U and late-onset BIBD.** The density of FUS inclusions throughout the brain and spinal cord were graded on a 3 point scale and then color coded as green (0, absent), green-yellow (0.5+, rare), yellow (1+, mild), orange (2+, moderate) and red (3+, severe). Pathology grades were used to generate topographical maps for (**A**-**C**) aFTLD-U and (**D**-**E**) BIBD cases. Brain regions are labeled as follows: 1 frontal cortex, 2 orbitofrontal cortex, 3 primary motor cortex, 4 primary sensory cortex, 5 superior and middle temporal cortex, 6, parietal cortex, 7 occipital cortex, 8 cingulate gyrus, 9 midbrain, 10 pons, 11, medulla, 12 spinal cord, 13 dentate nucleus, 14 amygdala, 15 entorhinal cortex, 16 anterior striatum, 17 globus pallidus, 18 posterior striatum, 19 thalamus, 20 subthalamic nucleus, 21 substantia nigra, 22 hippocampus, 23 dorsal medulla, 24 inferior olive, 25 cerebellum.

To visualize the distribution of FUS inclusions, their relative abundance was graded on a scale of 0 (absent) to 3 (severe) in all CNS regions examined here to generate pseudocolored topographical maps of the burden of FUS pathology in each case (Figure 2). All three cases of aFTLD-U showed a similar distribution of FUS pathology with prominent involvement of frontal and temporal cortex, hippocampus, parahippocampal gyrus, orbitofrontal cortex, cingulate gyrus, basal ganglia and midbrain (Figure 2A-C). In contrast, the topography of the FUS pathology in the two BIBD cases appeared different (Figure 2D-E) with prominent involvement of the pyramidal motor system (primary motor cortex, spinal cord) and extrapyramidal motor areas (inferior olive, basis pontis, substantia nigra, locus ceruleus, red nucleus, dentate nucleus of the cerebellum, globus pallidus, striatium, subthalamic nucleus, thalamus). Case B1 showed limited FUS pathology in neocortical regions (Figure 2D) while case B2 showed no neocortical FUS pathology (Figure 2E). Limbic regions including the parahippocampal gyrus, amygdala and hippocampus exhibited rare to no FUS pathology. These topographical maps were generated using a composite score for both NCIs and NIIs. An analysis of neocortical regions based solely on the density of NCIs yielded similar results in that the three cases of aFTLD-U exhibited more neocortical NCIs than the two BIBD cases, and so the difference in FUS inclusion density was not due to differences in inclusion type or localization (data not shown). Again, the near absence of NIIs in the two cases of BIBD contrasted starkly with the NIIs in multiple brain regions in aFTLD-U.

**Figure 3:**
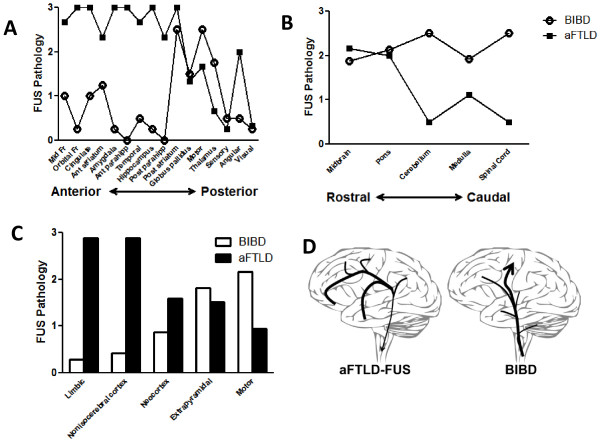
**Neuroanatomic distribution of FUS pathology in aFTLD-U and late-onset BIBD.** (**A**) FUS pathology grades for brain regions were averaged for aFTLD-U versus BIBD and forebrain regions were plotted from anterior brain regions (mid-frontal and orbitofrontal cortex) to posterior brain regions (visual cortex). aFTLD-U scores are shown with black squares while BIBD scores are shown with open circles. (**B**) Average FUS pathology grades for brainstem and spinal cord regions were plotted from rostral to caudal. Cerebellum scores represent grades from the dentate nucleus only, and does not reflect the absence of pathology in the cerebellar folia. aFTLD-U scores are shown with black squares while BIBD scores are shown with open circles. (**C**) Average FUS pathology grades for various groups of brain regions are shown for aFTLD-U (black bars) and BIBD (clear bars). Limbic regions included amygdala, hippocampus, parahippocampal gyrus, orbitofrontal cortex and cingulate gyrus. Nonisocerebral cortex regions included parahippocampal gyrus, cingulate gyrus and orbitofrontal cortex. Neocortical regions included mid-frontal cortex, superior and inferior temporal cortex, angular gyrus, motor cortex, sensory cortex and visual cortex. Extrapyramidal motor regions included basal ganglia, thalamus, midbrain including substantia nigra, pons including locus ceruleus, dentate nucleus of the cerebellum and the medullary inferior olive nucleus. Motor regions included the motor cortex and the ventral spinal cord grey matter. (**D**) The hypothetical spread of FUS pathology is shown based on the topography of FUS inclusions which emphasizes the more widespread involvement of the brain in aFTLD-U with a predilection for anterior forebrain, cerebral cortex and limbic brain regions compared to the pattern of FUS pathology in BIBD which involves primarily pyramidal and extrapyramidal motor regions with relative sparing of other brain regions. Although speculative, the topographic distribution of FUS pathology suggests that aFTLD-U and late-onset BIBD exhibit differences in the spread of FUS pathology.

To further explore the spatial topography of FUS pathology and with the caveat that very few cases were available for study, pathology grades for each brain region were averaged for aFTLD-U or BIBD and values for the forebrain were plotted from anterior to posterior (Figure 3A). aFTLD-U demonstrated a gradient of FUS inclusions with greater pathology involving anterior cerebrum relative to posterior cerebrum. Notably, this effect was not only due to the predilection for frontal and temporal cortex or due to the relative sparing of the visual cortex. For example, more anterior deep grey structures (caudate, globus pallidus) were more severely affected than the thalamus. In contrast, BIBD demonstrated a spatially restricted pattern with the most pathology towards the center of the anterior-posterior axis (namely the motor cortex, basal ganglia and thalamus) with relative sparing of the more anterior and posterior regions of the cerebrum.

To further explore the spatial distribution of FUS pathology, average pathology scores for the brainstem and spinal cord were plotted along the rostral to caudal axis (Figure 3B). aFTLD-U showed a predilection towards FUS pathology in the rostral brainstem with less FUS pathology caudally. In contrast, BIBD showed uniform FUS pathology along the entire rostral to caudal axis.

Finally, functionally similar brain regions were defined which relate to the clinical symptoms seen in FTD and MND, namely limbic regions, cerebral cortex (non-isocerebral cortex versus neocortex), extrapyramidal motor regions, and the pyramidal motor system. Average aFTLD-U versus BIBD FUS pathology scores were determined for these functionally distinct brain regions to determine whether aFTLD-U and BIBD exhibits differential involvement of these select anatomic areas (Figure 3C). aFTLD-U showed the most FUS pathology in limbic regions (amygdala, hippocampus, parahippocampal gyrus, cingulate gyrus and orbitofrontal cortex), followed by non-isocerebral cortex (allocortex, periallocortex and proisocortex), neocortex, extrapyramidal motor regions (striatum, globus pallidus, thalamus, midbrain/substantia nigra, pons, locus ceruleus, dentate nucleus of the cerebellum, medullary olive), and motor regions (primary motor cortex, ventral spinal cord). BIBD showed the opposite rank order in terms of FUS pathology, with greatest involvement of the pyramidal motor system followed by the extrapyramidal motor regions, neocortex, non-isocerebral cortex, neocortex and limbic regions.

## Discussion

FUS is one of three RNA- and DNA-binding proteins that comprise the FET protein family [20]. Together with EWS and TAF15, these FET proteins share structural similarities including the propensity to aggregate, and they all are components of fusion oncogenes associated with sarcomas and leukemias [20-22]. FUS is predominantly nuclear but does shuttle between the nucleus and cytoplasm, and is known to bind to a large number of RNA molecules to regulate mRNA splicing and stability [23-28]. Mutations in the FUS gene cause rare forms of ALS (ALS-FUS) in which motor neuron degeneration is associated with FUS immunoreactive inclusions [8,18]. There is heterogeneity of FUS pathology in ALS-FUS depending either on the type of *FUS* mutation and/or age of onset, ranging from round basophilic FUS positive NCIs to tangle-like FUS positive NCIs together with glial cytoplasmic inclusions [10]. Pathogenic mutations most often occur within conserved regions of exon 15 around the area that encodes a nuclear localization signal, and these mutations affect the binding of FUS to transportin 1, a protein known to facilitate the shuttling of various RNA-binding proteins including FET proteins into the nucleus, suggesting that the abnormal localization of FUS is linked to neurodegeneration [29-33].

There is a known clinical and pathologic overlap between ALS and FTD, and this overlap is demonstrated by the fact that various forms of FTLD including aFTLD, BIBD and NIFID exhibit FUS immunoreactive inclusions [14-16]. Unlike the inclusions seen in ALS-FUS, the FUS immunoreactive inclusions in these sporadic forms of FTLD are also immunoreactive for EWS, TAF15 and transportin 1 as reported elsewhere [34-37] and as described here. The neuropathology of FTLD-FUS is also heterogeneous but is clearly distinct from ALS-FUS due to increased diversity of inclusion morphology such as the presence of vermiform NIIs in aFTLD-U and NIFID, and a broader anatomic distribution of FUS pathology [10,11]. In addition to being distinct from ALS-FUS, detailed neuropathologic reports indicate that there are features which may be used to distinguish between the three FTLD-FUS subtypes, supporting the idea that aFTLD, BIBD and NIFID are related but distinct entities [5,9,11]. In particular, the presence of vermiform NIIs in aFTLD-U and the near absence of NIIs is BIBD appears to be one feature which can be used to distinguish aFTLD-U from BIBD [11].

Many neurodegenerative diseases show a stereotyped neuroanatomic progression of pathology including tau protein in AD, amyloid plaque pathology in AD and α-synuclein pathology in Parkinson’s disease (PD) [38-40]. While the number of cases studied worldwide is too small to make definitive judgments of the pathologic spread of FUS pathology, the few cases presented here suggest that the topography of FUS pathology in aFTLD-U is distinct from the topography of FUS pathology in late-onset BIBD. aFTLD-U pathology is most severe in anterior limbic and neocortical regions with a gradual diminution of pathology along the anterior-posterior and rostral-caudal axes (Figure 3D). In contrast, late-onset BIBD shows the most severe FUS pathology in pyramidal and extrapyramidal motor regions with FUS pathology involving the spinal cord, brainstem and deep grey structures, and relative sparing of neocortex (aside from motor cortex) and limbic areas (Figure 3D). Although additional cases are inevitably required to extend the findings reported here on these rare forms of FTLD, our findings suggest that there is a clinical and pathologic spectrum of FUS disease with a common molecular pathology (i.e. FUS inclusions), but with rather disparate pathologies both in terms of the subcellular localization and the neuroanatomic distribution of FUS inclusions.

The clinical manifestations of FUS pathology are varied, ranging from pure FTD to pure MND [5,9,11,14-17]. The two BIBD cases presented here were diagnosed with ALS-plus syndrome in which MND is associated with one or more clinical phenomena (i.e. dementia, parkinsonism) that have been considered by some to exclude the diagnosis of ALS [41-45]. However, it is now accepted that 10% of ALS patients manifest clinical features of FTD and up to 50% have measureable frontotemporal cognitive deficits [46]. Since reported cases of BIBD represent a spectrum ranging from pure MND to pure FTD, categorizing all BIBD cases as a subtype of FTLD-FUS does not seem to reflect the clinical and pathologic features of BIBD, particularly given that clinical signs of frontotemporal dysfunction may not be seen and frontal or temporal lobe degeneration may be absent. Better understanding of the molecular events underlying inclusion formation, and the downstream consequences of FUS abnormalities will help determine the similarities and disparities which define these heterogeneous diseases.

The three cases of aFTLD-U presented here are similar to those described by previous clinical and pathologic case series and reports of aFTLD-U [9,11,15,17]. In contrast, the two cases of BIBD presented here are clinically and pathologically unique. Most reported cases of BIBD exhibit an earlier onset of disease and a more widespread neuroanatomic distribution of FUS pathology [3-6,11,13,14]. The largest reported series of BIBD with FUS pathology included a total of 8 cases with an average age of onset of 46 years (ranging from 29 to 57) and an average age of death of 53 years (ranging from 39 to 68) [11,14]. This series included five cases of dementia (four of which were behavioral variant of FTD), two cases of ALS without dementia, and one case of progressive supranuclear palsy-like parkinsonism without dementia. These cases, together with nearly all additional published BIBD cases, exhibit a wide neuroanatomic distribution of FUS pathology including moderate to numerous numbers of NCIs in nearly every brain region analyzed including the frontal cortex and hippocampus. NIIs were absent to rare, and NCIs exhibited a wide variety of morphologies including crescentic, annular, granular, round, tangle-like and irregular. In contrast, the two BIBD cases reported here represent extreme examples in terms of age of onset (65, 75) and age of death (72, 78). The predominant clinical phenotype of these two BIBD cases was that of MND. Although NCI morphology was similar to that already reported for BIBD, the near absence of FUS pathology in limbic regions including the hippocampus and parahippocampal gyrus, and the relative sparing of cerebral cortex is not typical of BIBD.

Only two additional cases of BIBD without hippocampal FUS inclusions have been reported, [4,13] and they were clinically diagnosed with pure ALS without cognitive or exptrapyramidal features, while also being the two oldest cases of BIBD in the literature with age of onset at 73 and 75 (age of death of 75 and 79). The first case was reported as grossly unremarkable with microscopically evident motor neuron degeneration in association with basophilic inclusions [4]. FUS immunoreactive inclusions were observed in pyramidal and extrapyramidal motor systems including the anterior horn of the spinal cord, substantia nigra, oculomotor nuclei, red nuclei, inferior olivary nuclei, facial nuclei, pontine nuclei, dentate nuclei, hypoglossal nuclei, vestibular nuclei and locus coerulei [4]. The second case showed no gross evidence of cerebral atrophy, microscopically evident motor neuron degeneration, histologically evident basophilic inclusions, an absence of NIIs, and abundant round FUS immunoreactive NCIs involving the brainstem, spinal cord, thalamus and dentate nucleus [13]. The anatomic distribution of FUS pathology in these two late-onset BIBD cases are similar to the two cases presented here, including the predilection for pyramidal and extrapyramidal motor regions, the paucity of limbic and neocortical pathology, and sparing of the hippocampus. Indeed, the two previously reported cases and the two current cases are the four oldest cases of BIBD in the literature. Given that only four cases (including the two presented in the current study) of late-onset BIBD have been described, it remains to be seen whether old age at onset is a definite feature of BIBD cases in which FUS pathology preferentially affects motor regions with relative sparing of cerebral cortex and limbic structures. The MND-predominant clinical phenotype and the distribution of FUS pathology in these four cases of late-onset BIBD are somewhat similar to the clinicopathologic features of ALS-FUS. However, key distinctions between ALS-FUS and the late-onset cases of BIBD is the generally earlier onset of ALS-FUS, the absence of FUS mutation in late-onset BIBD, and the co-aggregation of FUS, EWS, TAF15 and transportin 1 in late-onset BIBD [3,6-8,10,18,35-37].

Care should be taken not to overanalyze data based on a limited number of cases. In particular, our analysis in which pathology scores were averaged according to histopathologic diagnosis was used as a means to understand the differences between the three cases of aFTLD-U and the two cases of BIBD reported here. As such, this analysis may not reflect the diversity or uniqueness of aFTLD-U and BIBD in general. For example, there is a possibility of selection bias in that the two cases of BIBD were phenotypically characterized by motor neuron disease and therefore may not be representative of BIBD in general which is phenotypically diverse. BIBD cases with motor neuron disease tend to show degeneration of motor systems (as observed in our cases) while BIBD cases with frontotemporal dementia show severe degeneration in the frontal cortex [11,14,47-52]. Although the density of FUS inclusions does not necessarily correlate precisely with the degree of neurodegeneration, we observed fewer neocortical FUS inclusions in BIBD compared to aFTLD-U, in contrast with larger case series which reported more FUS inclusions in the frontal cortex of BIBD relative to aFTLD-U which may reflect the broader clinical spectrum captured in previous case series. The clinical and pathologic heterogeneity of BIBD is remarkable and the two cases of BIBD reported here appear to represent one end of the spectrum of BIBD. Clearly, further experience is required to better understand this diversity. However, despite the issues associated with the low number of cases (representing <0.4% of the 1500+ cases in the CNDR Brain Bank), our analysis of these cases serves to highlight the diversity of FUS inclusion body diseases.

## Conclusions

We report here that the morphology and topography of FUS inclusions in aFTLD-U versus late-onset BIBD appear to be distinct. The rarity of these cases makes definitive judgment about the spatial topography or the staging of FUS neuropathology difficult. However, the spatial topography of AD and PD pathology supports the notion that inclusions form and progressively spread in a predictable neuroanatomic sequence. Experimental evidence has demonstrated that diverse neurodegenerative disease misfolded proteins and the inclusions they form can be propagated by direct inoculation of misfolded proteins, apparently via cell to cell transmission [53-62]. With the exception of prion diseases, this cell to cell transmission appears not to be infectious, at least for AD, PD and FTLD-Tau [63], but rather may regulate the spatial distribution and spread of pathology. FUS pathology in aFTLD-U and BIBD is topographically heterogeneous, but largely affects the brain regions linked to symptomatology, suggesting that the clinical phenotype in these diseases is dictated in part by differential spread of FUS pathology. Although the molecular basis for the pathologic diversity of sporadic FUS inclusion body diseases is entirely unknown, the clinical and pathologic features of the cases presented here suggest that late-onset BIBD may represent a unique or extreme example of sporadic MND within the diverse spectrum of FUS proteinopathies.

## Methods

### Clinical data

Brief summaries of the clinical features of each patient studied here are provided in Table 1.

### Neuropathologic analyses

Neuropathologic analysis was performed according to the standardized procedures of the Center for Neurodegenerative Disease Research (CNDR) Brain Bank at the University of Pennsylvania as previously described [64]. Briefly, brain and spinal cord regions were fixed in neutral buffered formalin, and 6 μm thick sections were cut from paraffin-embedded tissue. CNS tissue samples were obtained from the following regions for study here: mid-frontal cortex, orbitofrontal cortex, primary motor cortex, primary sensory cortex, superior and middle temporal cortex, parietal cortex (angular gyrus), occipital (primary visual) cortex, anterior cingulate gyrus, amygdala with parahippocampal gyrus, anterior striatum, posterior striatum with globus pallidus, thalamus, hippocampus with parahippocampal gyrus, cerebellum including dentate nucleus, midbrain including substantia nigra, pons including locus ceruleus, medulla including inferior olive and cervical spinal cord. Thoracic, lumbar and sacral spinal cord was also examined for BIBD cases.

Closely adjacent series of sections from each CNS region were stained with hematoxylin and eosin, thioflavin S, and Kluver-Barrera methods as well as by immunohistochemistry (IHC) using standard ABC methods with microwave antigen retrieval and a mouse monoclonal anti-FUS antibody (ProteinTech, Chicago, IL). We also confirmed the presence of FUS pathology in multiple additional sections using a rabbit polyclonal anti-FUS antibody (rabbit anti-FUS, Sigma Aldrich, St. Louis, MO). Microscopic slides were examined and the extent of FUS pathology was rated for each region on an ordinate scale (0 absent, 0.5 rare, 1 mild, 2 moderate and 3 severe) using previously described criteria for FUS pathology [11]. Inclusions were further characterized by IHC using antibodies specific for ubiquitin (1510, Chemicon, Temecula, CA), transportin 1 (D45, Sigma), TAF15 (rabbit anti-TAFII68, Bethyl Laboratories) and EWS (G-5, Santa Cruz Biotechnology, Santa Cruz, CA). IHC also was performed with additional antibodies including NAB228 (anti-Aβ), [65] SYN303 (anti-α-synuclein), phospho409/410-specific anti-TDP43 (1D3), [66] PHF1 (anti-phospho-tau) and RMO24 (anti-phospho-neurofilament-H) to exclude the presence of other neurodegenerative diseases including Alzheimer’s disease (AD), synucleinopathies, FTLD-TDP, FTLD-Tau and NIFID.

Neuropathologic criteria for the diagnosis of BIBD included the presence of numerous basophilic inclusions in a wide neuroanatomic distribution on H&E stained sections which were immunoreactive for FUS protein. The diagnosis of aFTLD-U was based on the presence of ubiquitin and FUS positive inclusions which could not be seen on H&E sections, and the paucity of basophilic inclusions on H&E stained sections. No cases of NIFID were found in the CNDR Brain Bank based on the absence of at least moderate numbers of neurofilament immunoreactive inclusions in cerebral neocortex or limbic regions.

### Genetic analyses

DNA was extracted from brain tissue using QIAsymphony DNA Mini Kit (Qiagen) following the manufacturer’s protocol. Standard Sanger sequencing was used to evaluate the entire coding region of *FUS* and adjacent intronic regions for cases B1 and B2, and mutation hot spot exons 14 and 15 for cases F1-3 (Beckman Coulter Genomics, Danvers, MA). Primers are available on request. Data were analyzed using Mutation Surveyor software (Softgenetics, State College, PA).

### Consent

The University of Pennsylvania Institutional Review Board has reviewed the CNDR Neurodegenerative Disease Autopsy Brain Bank protocols and has confirmed that these studies do not meet the criteria for human subjects research because according to 45 CRF § 45.102(f), a human subject is defined as a living individual. However, all patients reported here were pre-consented for autopsy prior to death including the use of tissues for scientific study. At time of death, consent for autopsy was reobtained from next-of-kin.

## Abbreviations

AD: Alzheimer’s disease; aFTLD-U: Atypical frontotemporal lobar degeneration with ubiquitin-positive inclusions; ALS: Amyotrophic lateral sclerosis; ALS-FUS: Amyotrophic lateral sclerosis with *FUS* mutations; ALS-plus: Amyotrophic lateral sclerosis-plus syndrome; BIBD: Basophilic inclusion body disease; CNS: Central nervous system; DN: Dystrophic neurites; EWS: RNA-binding protein EWS; FET: FUS, EWS, TAF15 family; FUS: Fused-in-sarcoma; FTD: Frontotemporal dementia; FTLD: Frontotemporal lobar degeneration; FTLD-FUS: Frontotemporal lobar degeneration with FUS inclusions; H&E: Hematoxylin and eosin; IHC: Immunohistochemistry; MND: Motor neuron disease; NCI: Neuronal cytoplasmic inclusions; NIFID: Neuronal intermediate filament inclusion body disease; NII: Neuronal intranuclear inclusions; PD: Parkinson’s disease; TAF15: TATA box binding protein-associated factor 15; TDP-43: Tar DNA binding protein 43.

## Competing interest

The authors declared that they have no competing interest.

## Authors’ contributions

EBL conceived and designed the study, performed neuropathologic assessments, analyzed and interpreted data and drafted the manuscript. JR performed immunohistochemistry, analyzed and interpreted data and revised the manuscript. HJ performed immunohistochemistry, and analyzed and interpreted data. LBE, LMC, DK, BLM, HBC and LFM performed clinical assessments, organized or summarized clinical data and reviewed the manuscript. JQT performed neuropathologic assessments and revised the manuscript. VMVD performed genetic analyses and revised the manuscript. All authors read and approved the final manuscript.

## Authors’ information

EBL is an Assistant Professor in the Department of Pathology & Laboratory Medicine at the University of Pennsylvania, the principle investigator of the Translational Neuropathology Research Laboratory in the Perelman School of Medicine at the University of Pennsylvania (http://www.med.upenn.edu/tnr/index.shtml), a co-investigator of the University of Pennsylvania CNDR Neurodegenerative Disease Brain Bank, and a practicing neuropathologist.
